# Predictors of Long-Term Relapse in Primary Monosymptomatic Nocturnal Enuresis: A Retrospective Cohort Study

**DOI:** 10.3390/children13010103

**Published:** 2026-01-10

**Authors:** Serap Ata, Sevim Yener

**Affiliations:** 1 Department of Pediatrics, Ümraniye Training and Research Hospital, University of Health Sciences, 34764 Ümraniye, Türkiye; 2 Department of Pediatric Urology, Ümraniye Training and Research Hospital, University of Health Sciences, 34764 Istanbul, Türkiye; sevim.yener2@saglik.gov.tr

**Keywords:** primary monosymptomatic nocturnal enuresis, relapse, functional bladder capacity, voiding diary, uroflowmetry, children

## Abstract

**Highlights:**

**What are the main findings?**

- Reduced functional bladder capacity (FBC/EBC ratio) based on the voiding diary was identified as the strongest predictor of long-term relapse in children with PMNE.
- Age, sex, family history, treatment modality (alarm, desmopressin, or combination therapy), and reduced UFM-derived bladder capacity showed no significant association with long-term relapse.

**What are the implications of the main findings?**

- A diary-based FBC/EBC ratio above 79% reliably indicates a low risk of long-term relapse and may assist clinicians in planning individualized follow-up strategies.
- Children with reduced diary-based bladder capacity may benefit from closer monitoring, prolonged maintenance therapy, or earlier supportive interventions to reduce the likelihood of relapse.

**Abstract:**

Introduction: Nocturnal enuresis is defined as involuntary urination during sleep in children, particularly those aged 5 years or older. Primary monosymptomatic nocturnal enuresis (PMNE) involves nighttime wetting without daytime symptoms, and although factors like reduced bladder capacity, nocturnal polyuria, and impaired arousal contribute, predictors of long-term relapse remain uncertain. Methods: This retrospective cohort study included 227 children aged ≥5 years with strictly defined PMNE who achieved complete remission following a standardized 3-month treatment protocol (alarm therapy, desmopressin, or desmopressin plus oxybutynin). All children underwent ICCS-based assessment, including physical examination, urinalysis, ultrasonography, UFM, a 48 h frequency/volume (F/V) diary, and post-void residual measurement. One year after treatment discontinuation, patients were reassessed using a 14-day wet-night diary. Predictors of relapse were analyzed using comparative statistics. Result: At 1-year follow-up, 48.5% of children experienced relapse. Age, sex, treatment modality, family history, and baseline wet-night frequency were not associated with relapse (*p* > 0.05). Diary-based FBC was significantly higher than UFM-based capacity (*p* < 0.001). Reduced diary-based mean FBC/EBC ratios were significantly more common among relapsing children (*p* < 0.001), whereas UFM-derived ratios showed no significant difference (*p* = 0.052). ROC analysis demonstrated moderate discriminatory performance for diary-based FBC/EBC (AUC 0.671). A ratio > 79% predicted sustained remission with 83.6% specificity and a positive predictive value of 73.5%. Conclusions: Diary-derived bladder capacity is the strongest predictor of long-term relapse in PMNE and outperforms UFM-based assessment. A mean FBC/EBC ratio > 79% provides a clinically useful threshold for identifying children at low risk of recurrence. Those with reduced diary-based capacity may benefit from closer follow-up or extended maintenance therapy.

## 1. Introduction

According to the International Children’s Continence Society (ICCS), nocturnal enuresis (NE) is defined as involuntary, intermittent bedwetting during sleep in children aged 5 years or older and is more common in males. The prevalence exceeds 10% at 6 years of age, decreases to approximately 5% at 10 years, and falls to 0.5–1% in adolescents and young adults [1,2]. Although the underlying pathophysiology is not fully understood, NE is considered a multifactorial condition resulting from the interaction of maturational delay, genetic predisposition, nocturnal polyuria, disturbed sleep, reduced bladder capacity, and detrusor overactivity [3]. Spontaneous resolution occurs in about 15% of affected children annually. NE is classified as primary when no sustained dry period of at least six months has ever been achieved, and secondary when bedwetting recurs after a dry period of six months or longer.

Primary monosymptomatic nocturnal enuresis (PMNE) is a common pediatric condition characterized by nighttime bedwetting in the absence of daytime lower urinary tract symptoms and is influenced by mechanisms such as nocturnal polyuria, reduced functional bladder capacity (FBC), and impaired arousal [1]. First-line management of PMNE includes education and counseling regarding the condition. In patients who do not benefit from initial approaches, alarm therapy, behavioral interventions, and pharmacological treatments become prominent options [2,3]. Current recommendations suggest that PMNE associated with nocturnal polyuria should be treated primarily with desmopressin, whereas PMNE characterized by reduced bladder capacity should be managed with alarm therapy as the first-line intervention. Anticholinergic agents such as oxybutynin are not theoretically indicated for routine PMNE treatment and should be considered only in a very small subgroup of patients with nocturnal overactive bladder during sleep [2].

Although alarm therapy, desmopressin, and combination treatments yield favorable short-term results, long-term relapse after discontinuation remains a major clinical challenge, with relapse rates varying widely across studies [4,5]. While factors such as age, baseline wet-night frequency, and bladder capacity have been identified as predictors of treatment response, the prognostic value of FBC remains inconsistent, largely due to methodological differences in bladder capacity assessment [6]. Functional bladder capacity may be reduced by up to 50% in children with nocturnal enuresis, with low age-adjusted FBC reported in 46.5% of patients and more commonly observed in those with daytime wetting and frequent nocturnal enuresis episodes [7]. Notably, many previous studies analyzed MNE and NMNE together, although reduced bladder capacity is an expected characteristic of NMNE due to the presence of underlying daytime lower urinary tract dysfunction, which may have confounded earlier findings [4,6]. In particular, the relationship between FBC measured by a 48 h voiding chart and FBC obtained via uroflowmetry (UFM) in predicting long-term relapse has not been clearly defined.

Therefore, this study aimed to determine one-year relapse rates among children with PMNE who achieved complete remission after treatment and to identify relapse predictors, with a specific focus on comparing bladder capacity measurements derived from voiding diaries and UFM.

## 2. Materials and Methods

### 2.1. Study Design and Patient Group

**Figure 1:**
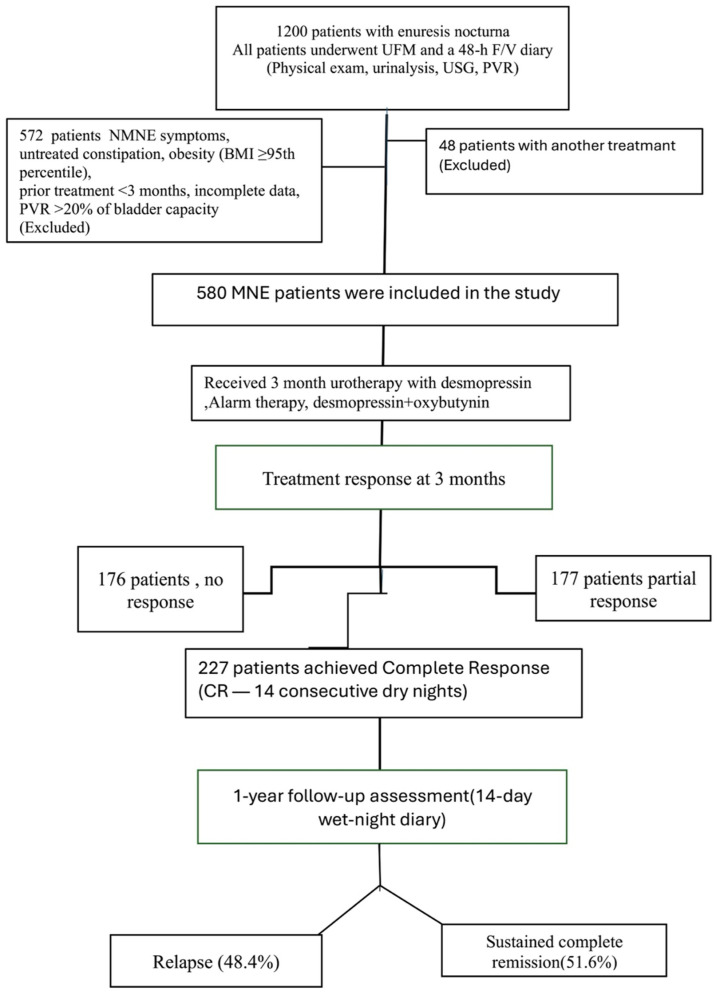
Patient flow chart.

The data in this study, including 227 patients, were obtained retrospectively from the data processing system of the Pediatric Urology and Pediatrics Clinics of Ümraniye Training and Research Hospital between January 2022 and May 2024. In our clinic, all children presenting with nocturnal enuresis undergo a standardized evaluation that includes a 48 h frequency/volume (48 h F/V) charts, uroflowmetry (UFM), and urinary tract ultrasonography. According to the International Children’s Continence Society (ICCS) definitions, monosymptomatic nocturnal enuresis (MNE) is characterized by nighttime bedwetting in children aged ≥5 years without any daytime lower urinary tract symptoms. Accordingly, none of the included children exhibited urgency, frequency, daytime incontinence, voiding postponement, infrequent voiding, dysuria, or weak urinary stream. Inclusion criteria were age ≥ 5 years, a confirmed diagnosis of PMNE, availability of complete (48 h F/V) charts and UFM data, documented post-void residual urine volume (PVR) assessment, receipt of alarm therapy, desmopressin, or combination treatment, and achievement of complete remission after a 3-month treatment course. Children with daytime lower urinary tract symptoms (NMNE), recurrent urinary tract infections, urinary or neurological disorders, untreated constipation, obesity (BMI ≥ 95th percentile), prior enuresis treatment within the preceding 3 months, incomplete data, protocol non-adherence, or a post-void residual exceeding 20% of functional bladder capacity were excluded from the study (Figure 1).

All children were evaluated in accordance with ICCS guidelines, including physical examination, urinalysis, urinary ultrasonography, a 48 h F/V charts, UFM, and post-void residual (PVR) assessment [8]. Seven-day nocturnal wetting records were completed both before and after treatment. Patients who achieved complete remission after three months of treatment were invited for a follow-up evaluation one year later, during which 7-day nocturnal wetting records were obtained again. All children underwent uroflowmetry and completed 48 h frequency/volume (48 h F/V) charts prior to treatment.

In accordance with ICCS recommendations, 48 h frequency/volume charts were used to assess bladder capacity, recording the timing and volume of each void as well as total fluid intake [8]. Voiding diaries were required to be completed for at least two consecutive days, and the maximum voided volume—excluding the first morning void—was included in the analysis, as the first morning void generally reflects overnight urine production [8].

At the initial visit, parents were thoroughly instructed by the physician on how to perform voiding measurements. All recordings were carried out during weekends under parental supervision. This approach was chosen because all enrolled children were of school age, and weekday recordings could be compromised by school attendance and parental work schedules. The rationale for this procedure was explained in detail to parents or caregivers during the clinical visit [8].

Bladder capacity was assessed using both the maximum voided volume derived from 48 h F/V charts and UFM-based measurements. Uroflowmetry was repeated when initial tracings were suboptimal, and abnormal flow patterns (tower, staccato, intermittent, or plateau) were excluded. Post-void residual urine (PVR) was interpreted according to age; incomplete bladder emptying may be physiological in infants and young children, whereas older children are expected to empty the bladder completely. A PVR volume greater than 20 mL was considered suggestive of abnormal or incomplete bladder emptying [1]. Maximum bladder capacity was calculated as the largest voided volume plus PVR [9]. Expected bladder capacity (EBC) was calculated using the ICCS formula (age × 30) + 30 mL [9], and capacities were classified as low (<65% EBC), normal (65–150%), or high (>150%), with distributions reported separately for chart-based and UFM-based measurements [8].

### 2.2. Treatment

All patients received standard urotherapy, which included regular daytime voiding, regulation of bowel habits, voiding before bedtime, and restriction of evening fluid intake. Families were thoroughly instructed on the use of alarm therapy, including the steps required to assist the child in awakening during the initial weeks of treatment. Patients were monitored every two weeks during the first month and subsequently once monthly up to the end of the third month. At each visit, a pediatric urologist evaluated both the child and the caregivers, provided reinforcement of urotherapy instructions, and contacted families by telephone if follow-up appointments were missed.

Pharmacologic treatment consisted of fast-release oral desmopressin. The initial dose was 120 µg, which was increased to 240 µg in non-responders, and gradually tapered during discontinuation. Combination therapy (desmopressin plus oxybutynin) was initiated in children who had previously used desmopressin without adequate response and who had been off all treatments for at least three months. Oxybutynin was administered at 0.2–0.4 mg/kg/day (maximum 0.4 mg/kg/day) for a total of three months and was tapered by alternate-day reductions during the discontinuation phase, while desmopressin therapy was continued concurrently.

Patients were categorized into three treatment groups: alarm (*n* = 24), desmopressin (*n* = 121), and desmopressin plus oxybutynin (*n* = 82). At the end of the 3-month treatment period, medications were discontinued using a structured tapering protocol (alternate-day dosing over a two-week period). Complete remission was defined as 14 consecutive dry nights, and treatment was stopped once this criterion was achieved. Treatment response was classified according to ICCS criteria: no response (NR), <50% reduction in wet nights; partial response (PR), 50–99% reduction; and complete response (CR), 100% reduction (full dryness).

Children who had achieved complete remission were instructed to return for evaluation if any symptoms recurred after structured discontinuation of therapy. However, those who did not present for follow-up during this period (*n* = 227) were contacted by telephone one year later and invited to the clinic for reassessment. None of these children had received any additional medication or sought care at another clinic during the interval. At this follow-up visit, patients were re-evaluated using a 14-day wet-night diary. Relapse was defined as the occurrence of more than one wet night per month after complete remission had been achieved [8]. Clinical and demographic characteristics were compared between children with and without relapse. Potential predictors of relapse included sex, age, family history, treatment modality, pre-treatment bladder capacity (48 h F/V charts and UFM-based), and pre- and post-treatment wet-night frequency.

### 2.3. Statistical Analysis

Statistical analyses were performed using SPSS version 27.0. The Kolmogorov–Smirnov test was applied to assess normality of the data distribution. Descriptive statistics (mean, standard deviation, median, minimum, maximum, frequency, and proportion) were reported. For within-group comparisons of non-parametric data, the Wilcoxon signed-rank test was used. Categorical variables were analyzed with the chi-square test and the Fisher–Freeman–Halton test, as appropriate. Receiver operating characteristic (ROC) curve analysis was also used to evaluate the discriminative ability of diary-based and UFM-derived bladder capacity parameters in predicting relapse. A two-sided *p* value < 0.05 was considered statistically significant.

## 3. Results

The mean age of the 227 patients (148 male, 79 female) was 9.12 ± 2.39 years (range: 5–18). The mean number of wet nights per week decreased significantly from 5.58 ± 0.78 before treatment to 2.1 ± 2.5 at the one-year follow-up (*p* < 0.001). Desmopressin monotherapy was administered to 53.3% of patients, desmopressin plus oxybutynin to 36.1%, and alarm therapy to 10.6% (Table 1).

The mean expected bladder capacity (EBC) was 298.5 ± 60.9 mL. Mean functional bladder capacity (FBC) measured using the 48 h F/V charts and UFM were 201.5 ± 105.4 mL and 168.9 ± 111.2 mL, respectively. 48 h F/V charts-based FBC values were significantly higher than UFM-based measurements (mean difference: 32.6 ± 110.9 mL; *p* < 0.001) (Table 2). The mean FBC in UFM/EBC ratio was decreased in 65.1%, normal in 31.7%, and increased in 3% of patients. According to the F/V chart, these proportions were 48.8% decreased, 51.1% normal (*p* < 0.001). Prior to treatment, 92% of all patients had severe nocturnal enuresis (>5 wet nights/week) [10]; however, baseline wet-night frequency was not associated with long-term treatment resistance (*p* = 0.368). One year after treatment, approximately 20% of patients still exhibited severe enuresis (Table 1).

A total of 48.5% of patients experienced relapse at the one-year follow-up. A comparative analysis of relapse and non-relapse groups showed no significant differences in age, sex, family history, treatment modality (alarm, desmopressin, or combination therapy), or baseline wet-night frequency (*p* > 0.05) (Table 3).

There were no significant differences among the between groups regarding decreased, normal, or increased FBC based on the mean UFM/EBC ratio (*p* = 0.052).

In contrast, the voiding diary-based bladder capacity (mean FBC/EBC ratio) demonstrated a strong association with relapse, with decreased FBC significantly more common in the relapse group compared with the non-relapse group (60.9% vs. 35.9%, *p* < 0.001) (Table 3).

ROC analysis showed that the diary-based the mean FBC/EBC ratio had a moderate ability to identify children who would remain relapse-free, with an AUC of 0.671 (95% CI: 0.605–0.731, *p* < 0.0001). Using the optimal cut-off value (>79%), the model achieved 42.7% sensitivity and 83.6% specificity, indicating that values above this threshold were more reliable for predicting sustained remission (PPV for no relapse: 73.5%).

**Figure 2:**
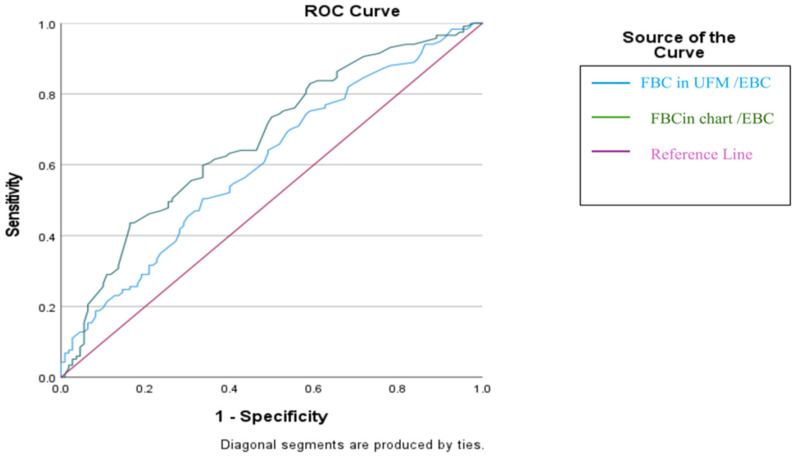
ROC Analysis of FBC/EBC Ratios.

Similarly, the UFM-derived mean FBC/EBC ratio demonstrated a lower performance in predicting no relapse, with an AUC of 0.606 (95% CI: 0.539–0.670, *p* = 0.0047). The >51% cut-off yielded 47.0% sensitivity and 67.3% specificity, suggesting a considerably weaker ability to identify children who would maintain remission compared with the diary-based measure (Table 4 and Figure 2).

In a stratified analysis of patients with complete remission, a diary-based FBC/EBC ratio >79% was consistently associated with a significantly lower risk of relapse across all treatment modalities, with a pooled Mantel–Haenszel odds ratio of 0.30 (95% CI: 0.16–0.55) and no evidence of heterogeneity between groups (*p* = 0.369) (Table 5).

## 4. Discussion

This retrospective cohort study evaluated clinical and bladder-capacity-related predictors of long-term relapse in children with primary monosymptomatic nocturnal enuresis (PMNE) who initially achieved complete response to treatment. The principal finding of this study is that functional bladder capacity (FBC) measured using the 48 h frequency/volume (F/V) diary was the strongest predictor of relapse one year after treatment discontinuation, whereas FBC derived from uroflowmetry (UFM) showed no meaningful predictive value. This distinction is clinically relevant, as relapse remains one of the major challenges in the long-term management of nocturnal enuresis despite the favorable short-term outcomes associated with alarm therapy, desmopressin, and combination regimens [2,9,10,11].

The diary-based mean FBC/EBC ratio showed a moderate but statistically significant ability to predict sustained long-term remission (AUC: 0.671; 95% CI: 0.605–0.731; *p* < 0.0001). Notably, the optimal cut-off value of >79% yielded high specificity (83.6%) and a positive predictive value of 73.5%, indicating that children above this threshold were substantially more likely to remain relapse-free. Although the International Children’s Continence Society (ICCS) defines reduced bladder capacity for diagnostic purposes as an FBC/EBC ratio < 65%, the diary-based mean FBC/EBC threshold of >79% identified in our study should be interpreted as a prognostic marker rather than a diagnostic criterion. This threshold primarily serves to identify children with PMNE who are at low risk of long-term relapse, rather than to exclude relapse risk.

While the diary-based FBC/EBC ratio demonstrated modest sensitivity, its high specificity indicates that this marker is particularly useful for confidently identifying children who are likely to remain relapse-free, rather than for capturing all children at risk of relapse. This supports its role in risk stratification and individualized follow-up planning.

Previous studies have reported an association between reduced FBC and treatment resistance, particularly in relation to poor response to desmopressin and increased need for anticholinergic therapy [2,12,13]. Our findings expand upon this concept by demonstrating that, even among children who initially respond to treatment, those with smaller diary-based bladder capacity are at substantially higher risk of losing remission over time. This suggests that FBC measured from voiding diaries is not only a marker of initial treatment response but also a valuable prognostic indicator of long-term outcomes.

A major limitation of much of the existing literature is the inclusion of mixed cohorts containing both MNE and NMNE. Because reduced FBC is an expected characteristic of NMNE due to underlying lower urinary tract dysfunction (LUTD), such heterogeneity obscures the true predictive role of bladder capacity [14]. In contrast, the present study focused exclusively on a strictly defined PMNE cohort with normal UFM patterns, normal PVR, and absence of daytime LUTS, thereby minimizing LUTD-related confounding and enabling a more accurate assessment of bladder capacity in the context of PMNE.

Another important observation was that diary-based FBC was significantly higher than UFM-based FBC (*p* < 0.001). This discrepancy has been noted previously and may reflect suboptimal bladder filling, rapid pre-test hydration, or voiding anxiety during hospital-based UFM assessment [15]. In contrast, the 48 h F/V diary captures habitual voiding patterns in a natural home environment and may therefore better represent true physiological bladder capacity. This likely explains why only diary-derived FBC, and not UFM-derived FBC, demonstrated a significant relationship with long-term outcomes.

Consistent with this observation, the UFM-derived FBC/EBC ratio showed weaker predictive performance (AUC: 0.606), with limited sensitivity and specificity, supporting the view that UFM should not be used as a standalone tool for long-term risk stratification but rather interpreted alongside diary-based assessments.

Demographic characteristics, including age, sex, family history, and baseline wet-night frequency, were not associated with relapse—consistent with prior large-scale cohorts Likewise, treatment modality (alarm therapy, desmopressin, or combination therapy) did not influence long-term outcomes once complete response was achieved. These observations underscore that baseline bladder capacity, rather than therapeutic modality or demographic factors, plays the pivotal role in determining relapse risk.

Although treatment allocation was not randomized, a stratified analysis limited to patients who achieved complete remission showed that the predictive value of a diary-based mean FBC/EBC ratio > 79% was consistent across alarm, desmopressin, and combination therapy groups, supporting its robustness as a treatment-independent prognostic marker.

The relatively high number of wet nights at baseline among children in our cohort, along with the persistence of two to three wet nights per week in 61% of relapsing cases at follow-up, may reflect the tertiary-care referral nature of our center and the increased severity of cases we encounter. Studies by Borg et al. provide additional context, demonstrating that many children exhibit markedly reduced nocturnal bladder capacity despite normal daytime Maximal voided volume (MVV), which may explain desmopressin failure even in the presence of adequate antidiuretic response [16]. Other investigations have also highlighted reduced FBC and increased bladder wall thickness on ultrasonography as potential modifiers of treatment response, particularly in relation to anticholinergic therapy [17,18,19].

Due to the retrospective design of our study, the exact timing of relapse could not be determined. van Kampen et al. reported that, following comprehensive treatment, overactive bladder was the only factor significantly associated with relapse in children with primary nocturnal enuresis and emphasized that frequent clinician–family contact may independently contribute to long-term treatment effectiveness. These findings suggest that both underlying bladder dysfunction and continuity of care should be considered when interpreting long-term outcomes [20].

## 5. Limitations

This study has several limitations. Its retrospective design introduces the risk of selection and recall biases. The study cohort consisted of children who achieved complete remission after a three-month treatment period but did not attend routine follow-up visits during the subsequent year. Consequently, the exact timing of relapse could not always be reliably determined, as families were often unable to provide clear information regarding when enuresis recurred. When parents were asked why they had not returned to the clinic despite the recurrence of symptoms, they commonly reported that they expected the condition to improve spontaneously over time or had lost confidence in the effectiveness of the medication or the overall treatment approach. Our center is the only pediatric urology clinic in the region, and patients generally return to our clinic for follow-up; however, this could not be fully confirmed for all cases. Adherence to urotherapy could not be objectively verified, and the socioeconomically disadvantaged characteristics of the study population may have influenced compliance. As this study was conducted in a tertiary referral center, the findings may not be fully generalizable to primary care or community-based populations, where relapse rates and bladder capacity distributions may differ; therefore, validation in less selected cohorts is warranted. Children who experienced relapse and were invited back to the clinic were restarted on appropriate treatment.

## 6. Conclusions

In conclusion, the diary-based mean FBC/EBC ratio represents a clinically meaningful prognostic marker for long-term outcomes in children with primary monosymptomatic nocturnal enuresis. A threshold value of >79% demonstrated high specificity and reliably identified children at low risk of relapse after achieving complete remission, offering a more precise prognostic indicator than the conventional ICCS diagnostic cut-off. Although its sensitivity was modest, the high specificity of this marker supports its utility for risk stratification and individualized follow-up planning. Routine assessment of bladder capacity using 48 h voiding diaries may therefore facilitate more tailored management strategies, allowing closer surveillance and prolonged maintenance therapy in high-risk children while avoiding unnecessary follow-up in those at low risk of relapse.

## Figures and Tables

**Table 1:** Baseline characteristics of the study population.

Age, years	Mean ± SD	9.12 ± 2.39
	Median (Min–Max)	9 (5–17)
Sex n (%)	Male	148 (65.2)
	Female	79 (34.8)
Family history, n (%)	No	101 (44.5)
	Yes	126 (55.5)
Wet nights/week (Before treatment)	Mean ± SD	5.58 ± 0.78
	Median (Min–Max)	6 (3–7)
Wet night/week (1 year after the treatment)	Mean ± SD	2.06 ± 2.45
	Median (Min–Max)	1 (0–7)
PVR (mL)	Mean ± SD	12.4 ± 6.8
	Median (Min–Max)	10 (0–20)
FBC in UFM (mL)	Mean ± SD	168.34 ± 111.20
	Median (Min–Max)	137 (39–751)
FBC in chart (mL)	Mean ± SD	201.52 ± 105.44
	Median (Min–Max)	195 (60–750)
FBC in UFM/EBC (%)	Mean ± SD	57.26 ± 35.95
	Median (Min–Max)	47 (11–196)
FBC in chart/EBC (%)	Mean ± SD	67.17 ± 30.11
	Median (Min–Max)	60 (16–160)
	Ratio	n (%)
FBC in UFM/EBC (%)	Decreased	148 (65.1)
	Normal	72 (31.7)
	Increased	7 (3.0)
FBC in chart/EBC (%)	Decreased	111 (48.8)
	Normal	116 (51.1)
Treatment, n(%)	Alarm	24 (10.6)
	Desmopressin	121 (53.3)
	Desmopressin + oxybutynin	82 (36.1)
1-year follow-up relapse		110 (48.5%)

EBC, expected bladder capacity; FBC in chart, functional bladder capacity in 48 h frequency/volume chart; FBC in UFM, FBC in uroflowmetry with post-void residual volume, PVR, post-void residual urine volume was measured by ultrasonography.

**Table 2:** Functional Bladder Capacity of Patients.

	Mean	SD	Median	Minimum	Maximum	*p*
FBC in chart	201.52	105.43	195.00	60.00	750.00	<0.001 *
FBC in UFM	168.94	111.20	137.00	39.00	751.00	
Bladder Capacity Difference	32.58	110.91	36.00	−386.00	507.00	

* Wilcoxon Signed-Rank Test, FBC in chart, functional bladder capacity in 48 h frequency/volume chart; FBC in UFM, FBC in uroflowmetry with post-void residual volume.

**Table 3:** Comparison of baseline characteristics between relapse and non-relapse groups.

Parameter	Category	Relapse (n = 110)	No Relapse (n = 117)	*p*-Value
Age	Mean ± SD	9.53 ± 2.27	9.58 ± 2.51	0.803
	Median (min–max)	9.1 (5.5–14.9)	9.1 (5.2–17.9)	
Sex	Male	71 (47.9%)	77 (52.1%)	^a^ 0.952
	Female	39 (49.4%)	40 (50.6%)	
Family history	No	53 (52.0%)	49 (48.0%)	^a^ 0.929
	Yes	57 (49.1%)	68 (50.9%)	
Treatment	Alarm	9 (37.5%)	15 (62.5%)	^b^ 0.345
	Desmopressin	57 (47.1%)	64 (52.9%)	
	Desmo + Oxybutynin	44 (53.7%)	38 (46.3%)	
Wet nights/week	Mean ± SD	5.65 ± 0.77	5.51 ± 0.79	^a^ 0.368
FBC in UFM/EBC (%) mean ± SD	Decreased	79 (71.8%)	69 (59%)	^b^ 0.052
	Normal	30 (27.3%)	42 (35.9%)	
	Increased	1 (0.9%)	6 (5.1%)	
FBC in chart /EBC) (%) mean ± SD	Decreased	67 (60.9%)	44 (35.9%)	^b^ <0.001 *
	Normal	43 (39.1%)	73 (62.4%)	

^a^ Chi-Square Test; ^b^ Fisher–Freeman–Halton Test, * *p*-value < 0.05.

**Table 4:** ROC-based diagnostic performance of diary- and UFM-derived FBC/EBC ratios.

	Diagnostic Scan					ROC Curve		*p*
	Cut Off	Sensitivity	Specificity	Positive Predictive Value	Negative Predictive Value	Area	95% Confidence Interval	
FBC in UFM/EBC	>51	47.01	67.27	60.4	54.4	0.606	0.539 to 0.670	0.0047
FBC in chart/EBC	>79	42.74	83.64	73.5	57.9	0.671	0.605 to 0.731	<0.0001

**Table 5:** Stratified analysis of relapse according to diary-based FBC/EBC ratio (>79%) across treatment groups in patients with complete remission.

Treatment Modality	Diary-Based FBC/EBC	Relapse (n/N, %)	Odds Ratio (95% CI)
Alarm	≤79%	7/11 (63.6)	Reference
	>79%	2/13 (15.4)	0.10 (0.02–0.73)
Desmopressin	≤79%	44/81 (54.3)	Reference
	>79%	13/40 (32.5)	0.40 (0.18–0.90)
Desmo + oxyb	≤79%	40/66 (60.6)	Reference
	>79%	4/16 (25.0)	0.22 (0.06–0.75)

Mantel–Haenszel pooled odds ratio: 0.30 (95% CI: 0.16–0.55), test for heterogeneity across treatment groups: *p* = 0.369.

## Data Availability

The datasets used and/or analyzed during the current study are available from the corresponding author on reasonable request.
